# Reducing the Gap in Knowledge and Expectations between Clinicians and People with Polycystic Ovary Syndrome or Adrenal Conditions: Simulation via Instant Messaging—Birmingham Advance: Patient and Public Involvement (SIMBA-PPI) Study

**DOI:** 10.1186/s12909-024-05772-w

**Published:** 2024-07-22

**Authors:** Eka Melson, Fatema Rezai, Carina Pan, Sung Yat Ng, Tamzin Ogiliev, Ella Blendis, Haaziq Sheikh, Harjeet Kaur, Catherine Cooper, Farah Abdelhameed, Francesca Pang, Shreya Bhatt, Dania Shabbir, Zahra Olateju, Eloise Radcliffe, Prashanthan Balendran, Abby Radcliffe, Gar Mun Lau, Meri Davitadze, Dengyi Zhou, Kashish Malhotra, Caroline Gillett, SIMBA and CoMICs team, Punith Kempegowda

**Affiliations:** 1 Institute of Clinical Sciences, Imperial College London, London, United Kingdom; 2 Institute of Metabolism and Systems Research, University of Birmingham, Birmingham, United Kingdom; 3 College of Medical and Dental Sciences, University of Birmingham, Birmingham, United Kingdom; 4 University of Lancaster, Lancaster, United Kingdom; 5 Walsall Manor Hospital, Walsall, United Kingdom; 6 Queen Charlotte’s and Chelsea Hospital, London, United Kingdom; 7 University of Warwick, Conventry, United Kingdom; 8 Jinnah Medical and Dental College, Karachi, Pakistan; 9 Clinic NeoLab, Tbilisi, Georgia; 10 Institute of Applied Health Research, University of Birmingham, Birmingham, United Kingdom; 11 Northwick Park Hospital, London North West University Healthcare NHS Trust, London, United Kingdom; 12 Rama Medical College Hospital, Hapur, Uttar Pradesh India; 13 Queen Elizabeth Hospital Birmingham, University Hospitals NHS Foundation Trust, Birmingham, Uttar Pradesh United Kingdom

**Keywords:** Patient and public involvement, Simulation-based learning, SIMBA, Medical education, Collaboration, Continuing professional development

## Abstract

**Background:**

To evaluate the efficacy of SIMBA as an educational intervention for both HCPs and people with either PCOS or adrenal conditions and to study the change in knowledge of people with PCOS or adrenal conditions about the conditions and expectations from the HCPs involved in their  care following SIMBA-PPI sessions.

**Methods:**

Two SIMBA-PPI sessions (SIMBA-PPI Polycystic ovary syndrome (SIMBA-PCOS) and SIMBA-PPI Adrenal conditions (SIMBA-Adrenal conditions)) were conducted in September 2021 and March 2022. In both sessions, HCPs interacted with moderators on patient management through WhatsApp. Patients with respective conditions underwent workshop-style learning in the same cases. SIMBA-PCOS transcripts were also translated into Brazilian Portuguese and workshops were held in both Brazilian Portuguese and English. The two groups (HCPs and patients) were then brought together to discuss exploring gaps in knowledge and expectations. The Wilcoxon Signed-Rank test compared differences in pre- and post-SIMBA self-reported confidence levels in HCPs and patients. Qualitative data from the online recordings were transcribed and analysed with inductive thematic analysis to identify gaps in knowledge and expectations from managing the cases.

**Results:**

48 HCPs and 25 patients participated in our study. When compared to pre-SIMBA confidence levels, SIMBA-PPI sessions effectively improved clinicians’ confidence in managing PCOS (40.5%, *p* < .001) and adrenal conditions (23.0%, *p* < .001) post-SIMBA. Patient participants’ confidence in HCPs significantly increased in the PCOS session (SIMBA-PCOS: 6.25%, *p* = 0.01).

**Conclusions:**

Integration of PPI into SIMBA improved HCPs' confidence in managing PCOS and adrenal conditions. SIMBA-PPI also improved patients’ confidence in HCPs. Our findings suggest that participating in SIMBA-PPI sessions can reduce the gap in knowledge and expectations between patients and HCPs involved in their care.

**Supplementary Information:**

The online version contains supplementary material available at 10.1186/s12909-024-05772-w.

## Introduction

Patient and public involvement (PPI) are essential for an equal partnership in clinical decisions and patient-centred care [1]. The General Medical Council, the regulatory body of doctors in the United Kingdom, first highlighted the importance of PPI in the standards for undergraduate education in “Tomorrow’s Doctors”, published in 2009 and later in the postgraduate ‘Standard for Deaneries’ calling for deaneries to ensure active and meaningful involvement of patients and the public in doctors’ training [2, 3]. Until then, patients’ role in education was indirect or passive, with limited contribution to curriculum development and its delivery and student assessment [4–6]. Yet, amidst this recognition, robust models that effectively integrate established educational theories into PPI initiatives within healthcare settings are lacking. There is a paucity of such activities in doctors’ postgraduate medical education. There is a lack of ontological and epistemological aspects and evidence of reproducibility, scalability, and sustainability in the published PPI models in medical education [7–9].

Despite the richness of educational theories such as Experiential Learning and Participatory Action Research [10], their application in developing comprehensive frameworks for PPI within medical education is limited (a brief explanation of all educational theories and frameworks referenced is supplied in Supplementary 1). While theories like the Health Belief Model [11] and Theory of Planned Behaviour [12] offer valuable insights into patient behaviours and attitudes, their integration into educational models specifically tailored for fostering patient-provider collaboration is sparse. The potential of the Transtheoretical Model to cater to diverse patient readiness levels for engagement in healthcare decisions remains underexplored. Similarly, empowerment theory [13] and social cognitive theory [14], which hold promise in cultivating empowered, informed patient partnerships, have yet to find their place in structured curricula to train future healthcare professionals. This scarcity of models utilising these theories in the context of PPI represents a critical gap in healthcare education. The dearth of comprehensive frameworks that integrate these well-established educational theories stifles the development of healthcare professionals capable of effectively collaborating with, learning from, and empowering patients and communities.

Simulation via Instant Messaging—Birmingham Advance (SIMBA) is a virtual simulation platform that successfully improved participating clinicians’ confidence in managing various simulated cases with a reasonable acceptance rate and reproducibility [15–18]. SIMBA sessions have been limited to healthcare professionals (HCPs) who underwent simulation-based learning through text messages in WhatsApp, followed by interactive discussions on Zoom. We recently conducted two educational sessions through SIMBA involving HCPs and people affected by PCOS or adrenal conditions. We called these sessions Simulation via Instant Messaging-Birmingham Advance Public and Patient Involvement (SIMBA-PPI). The two SIMBA-PPI sessions were SIMBA-PPI Polycystic ovary syndrome (SIMBA-PCOS) and SIMBA-PPI Adrenal conditions (SIMBA-Adrenal conditions).

The SIMBA-PPI model is based on the simulation game and Kolb’s experiential learning theory [19]. A simulation game is characterised by the outcomes of choices enacted by participants, interconnected within a framework of rules and resource references mirroring real-life scenarios [19]. David Kolb’s experiential learning theory is a model of human learning with 4 distinct stages [20]:

- Stage 1—concrete experience—is enacted by the simulation session whereby HCPs partake in case scenarios, and patients with PCOS and adrenal pathologies undergo the same cases in workshop-style learning
- Stage 2—reflective observation—is depicted by the post-simulation discussion with experts via Zoom. The HCPs and patients joined the same discussion, allowing a dialogue regarding bridging the knowledge gap between the two groups.
- Stage 3—abstract conceptualisation—was facilitated by the use of post-SIMBA surveys, which were distributed to all HCPs and patients partaking in SIMBA.
- Stage 4—active experimenting—is when HCPs and patients can apply their knowledge from the SIMBA simulation sessions to their own lives [20].

The aims of this study were:

1. To evaluate the efficacy of SIMBA as an educational intervention for both HCPs and people with either PCOS or adrenal conditions
2. To study the change in knowledge of people with PCOS or adrenal conditions about the conditions and expectations from the HCPs involved in their care following SIMBA-PPI sessions.

## Methods

The SIMBA-PCOS session was conducted on 21st September 2021, and the SIMBA-Adrenal conditions session was conducted on 8th March 2022. Both sessions were carried out based on integrating the six-step conceptual framework described by Kern et. al [21] and the Plan, Do, Study, Act (PDSA) cycle framework (Supplementary 2). This study has received ethics approval from the Science, Technology, Engineering and Mathematics Ethics Committee at the University of Birmingham (ERN_2023-0495). HCPs were recruited via advertisements on the SIMBA website and social media platforms (Facebook and Twitter). PPI participants were recruited through various support groups. Participation was entirely voluntary. Informed consent was taken from each participant.

### SIMBA-PCOS six-step conceptual framework and PDSA cycle 1

#### Problem identification and general needs assessment

PCOS commonly affects 10–20% of women worldwide [22]. Following the diagnosis, people with PCOS are often left with unmet information needs, which can further impact their mental health [23, 24]. This is probably due to the diverse symptoms of PCOS and the lack of awareness among HCPs and patients regarding the international consensus guidelines for managing people with PCOS [25]. Current educational interventions towards improving knowledge, attitudes, and practices of PCOS amongst HCPs have limited patient and public involvement. Further, there is a deeply rooted mistrust of HCPs among people with PCOS [24, 26]. The collaboration between patients and HCPs can effectively bridge the existing knowledge gap between these two groups. This collaborative approach may encourage greater patient engagement in their healthcare journey and potentially enhance educational initiatives to improve knowledge, attitudes, and practices related to PCOS among healthcare providers.

### Targeted needs assessment

We recognised the need for a model to increase understanding of their condition among people with PCOS. Our objective centred on reducing the knowledge gap between HCPs and patients, primarily due to the varied nature of PCOS symptoms, allowing HCPs and affected patients to engage in a dialogue.

### Goals and objectives

- Promote patient and public involvement in clinical decisions and care to achieve an equal partnership between healthcare professionals and patients,
- Involve people with PCOS in educational interventions to represent and support others,
- Integrate PPI into medical education at both undergraduate and postgraduate levels. This involves active and meaningful participation of patients and the public in doctors' training.

### Education strategies

The SIMBA-PPI model is based on the simulation game and Kolb’s experiential learning theory [19].

### Implementation of the educational model and PDSA Cycle 1

#### Planning cycle 1

This session was divided into two arms: one focused on HCPs with 29 participants and another component involving people with PCOS with 15 participants. The cases for SIMBA PCOS were designed based on PCOS clinical guidelines and a study on the learning needs of people with PCOS and HCPs [25, 27, 28]. These scenarios were converted into anonymised transcripts, with experts' inputs, which included information on past medical history, clinical examinations, laboratory investigations, and imaging results (an example transcript is provided for SIMBA PCOS and SIMBA Adrenal conditions as supplementary 4A-B).

#### Doing cycle 1

Moderators for the simulation were recruited through advertisements and trained in each simulated scenario. HCPs interacted with moderators on WhatsApp, taking relevant history and requesting appropriate investigations to make diagnoses and management/follow-up plans. The moderators provided the information requested by the HCPs from the pre-prepared transcripts. If the requested information was unavailable in transcripts, the moderator replied, 'Information requested is not available’. After completing all the simulated cases, participants attended a discussion about the cases with appropriate experts. We previously published a detailed description of the various steps involved in the SIMBA session, and a summary is included in Supplementary 5 [18].

In parallel, people with PCOS were invited to participate in workshop-style learning. Here, the transcripts of cases used in SIMBA PPI-HCP were presented to participants, sharing information on how people with PCOS were managed in real life. This was followed with a discussion of the instances concentrating on ‘what was done well’, ‘what could be done better’, and ‘what was not covered’ during the case management. A participant was invited to volunteer and summarise each case, which was presented to the other participants during the discussion.

##### Case discussion with Q&A

This session brought together HCPs and people with PCOS to discuss the cases in detail. Thirty minutes were allocated per case. In the first 5 min, experts presented the case to the group. This was followed by a discussion of the cases about evidence-based guidelines (5 min). In the following five minutes, HCPs who participated in the simulation asked their queries on the cases. The participant from the SIMBA PPI-PCOS group who summarised the discussions shared their findings on that case for five minutes. Each session was concluded with a 10-min discussion on the challenges of managing the cases to identify the gap in knowledge and expectations between people with PCOS and their HCPs. In total the case discussion lasted 2.5 h.

### Evaluation and feedback

#### Studying cycle 1 and Acting Cycle 1

We invited feedback from the SIMBA team and the expert chairs of the session. This was combined with the feedback from HCPs and PPI participants. The summary of the feedback is as follows which was implemented:

1. SIMBA team members suggested a serial instead of a parallel approach for HCP and PPI sessions to better distribute the workload.
2. Chairs recommended a pre-session meeting instead of just emails to understand the session better.
3. The main feedback from HCP participants was to conduct a session on various cases.

PPI participants recommended either breaking down the cases over multiple sessions or reducing the number of cases to allow for more in-depth exploration enabling equal participation from all attendees and ensure representation from a broader range of patient groups.

### SIMBA-Adrenal conditions six-step conceptual framework and PDSA cycle 2

#### Problem identification and general needs assessment

In contrast, compared to PCOS, most adrenal conditions are classified as rare diseases due to their low prevalence [29, 30]. However, if they remain unrecognised, patients can develop adrenal crisis, which can be fatal. Therefore, HCPs must establish effective educational interventions to recognise adrenal conditions early on. Involving those with adrenal conditions can help HCPs improve their communication skills in the diagnosis and management of adrenal conditions. In this session, we considered all the suggestions from the SIMBA-PCOS session and worked to optimise the model to address better our aim of bridging the knowledge gap between patients and HCPs.

### Targeted needs assessment

We identified the need for a model to accommodate better the HCPs and patients participating in the SIMBA PPI study. Instead of both HCP and PPI arms in parallel, people with adrenal conditions were invited on a separate day beforehand. This was implemented following feedback from the SIMBA team members, as detailed above. It also provided more options for PPI members, enabling them to arrange the session at a time of their convenience instead of a fixed slot. This also allowed the SIMBA team more time to summarise the discussions of each case beforehand.

Furthermore, we created an opportunity for input from Brazilian Portuguese-speaking communities. To facilitate this, the six case transcripts were translated into Brazilian Portuguese. A SIMBA team member fluent in English and Brazilian Portuguese held the workshop-style learning in Brazilian Portuguese. They transcribed and summarised the discussion from the workshop into English, which was presented at the discussion.

### Goals and objectives

We aimed to develop a model that promoted PPI in clinical decisions and allowed an open dialogue between patients and HCPs, improving the clinical care of people with adrenal conditions.

#### Education strategies

As with SIMBA-PCOS, the SIMBA-Adrenal conditions model is based on the simulation game and Kolb’s experiential learning theory [19].

### Implementation of the educational model and PDSA Cycle 2

#### Planning cycle 2

For the session, the cases included Addison’s disease, adrenal incidentaloma, adrenocortical carcinoma, Cushing’s syndrome, mild autonomous cortical secretion, and primary aldosteronism. Instead of both HCP and PPI arms in parallel, people with adrenal conditions were invited on a particular day beforehand. This was implemented following feedback from the SIMBA team members, as detailed above. The rest of the planning was similar to the first cycle, where PCOS scenarios were simulated.

#### Doing cycle 2

27 HCPs underwent simulation-based learning focusing on six case scenarios of adrenal conditions as detailed in the SIMBA-PCOS session. PPI workshops were held a week before the SIMBA-Adrenal conditions session, and conducted similar to SIMBA-PCOS session.

##### Case discussion with Q&A

This session brought together HCPs and people with PCOS to discuss the cases in detail. Thirty minutes were allocated per case. In the first 5 min, experts presented the case to the group. This was followed by a discussion of the cases about evidence-based guidelines (5 min). In the following five minutes, HCPs who participated in the simulation asked their queries on the cases. The participant from the SIMBA PPI-PCOS group who summarised the discussions shared their findings on that case for five minutes. Each session was concluded with a 10-min discussion on the challenges of managing the cases to identify the gap in knowledge and expectations between people with PCOS and their HCPs. In total the case discussion lasted 2.5 h.

##### Case discussion with Q&A

We introduceds several changes to the case discussion compared to the first cycle. ItWe extendeds the total duration to 3.5 hours, compared to 2.5 hours in the first version, by increasing the time allocated per to ensure each case discussion lasted to 30 minutes. NotablyIn contrast to cycle one, each case discussionit beginsan with a 10-minute pre-recorded expert talk to avoid technical issues and save time, unlike the live 5-minute expert presentation in the first version. Additionally, a SIMBA PPI-Adrenal group member provides a 5-minute summary, and the session concludes with a longer, 15-minute open Q&A, involving all expert panel members, allowing for a more comprehensive discussion. These changes aim to streamline the process and enhance the depth of interaction between healthcare professionals and participants.

### Evaluation and feedback

#### Studying cycle 2

We gathered feedback from SIMBA team members, chairs, experts involved in the discussion, and participants. The primary feedbacks were:

1. SIMBA team members felt the session was much better regarding organisation and delivery than the SIMBA-PPI PCOS session.
2. Chairs recommended a follow-up email following the pre-session meeting to summarise the plans for the session.
3. Participants recommended to continue organising similar sessions but aim for weekdays instead of weekends.

#### Acting cycle 2

We gathered all the feedback and analysed the data from both sessions to recommend future sessions, as detailed in the results and discussion sections below.

### Data collection and statistical analyses

To answer our research questions, we used a mixed quantitative and qualitative research method. The analysis included data from participants who completed both pre- and post-SIMBA surveys.

#### HCP participants

Baseline characteristics, including location and level of training, were recorded. The differences in pre- and post-SIMBA questionnaire responses measured the changes in participants' self-reported confidence levels in managing cases included in the SIMBA-PPI session. The impact of participant’s responses to the six domains of medical education based on the core competencies of the Accreditation Council for Graduate Medical Education (ACGME) was also assessed [31]. Participants could select as many competencies as they found relevant to the session, including patient care, professionalism, knowledge of patient management, system-based practice, practice-based learning, and communication skills.

#### PPI participants

demographic information including age, ethnicity, location, and years since diagnosis were recorded. The efficacy of SIMBA in increasing knowledge of the respective conditions was explored through a series of questions and measured using a 5-point Likert scale. Pre- and post-SIMBA questionnaires were used to evaluate the efficacy (Supplementary 6A-D). These consisted of questions on knowledge regarding the conditions pre- and post-simulation. Unfortunately, due to the split between the workshop and the HCP simulation, many PPI participants did not attend the discussion session; hence, post-SIMBA questionnaires were not filled. The workshops and discussion sessions were recorded and transcribed via Zoom. The content of the transcripts was closely examined and analysed thematically by two independent study authors. Responses from the transcribed sessions were first read and familiarised, systematically identifying the text's main points and attaching labels/codes to capture the main ideas. Relevant and recurrent codes were then collated into themes inductively (data-driven themes) and reviewed.

**Fig. 1:**
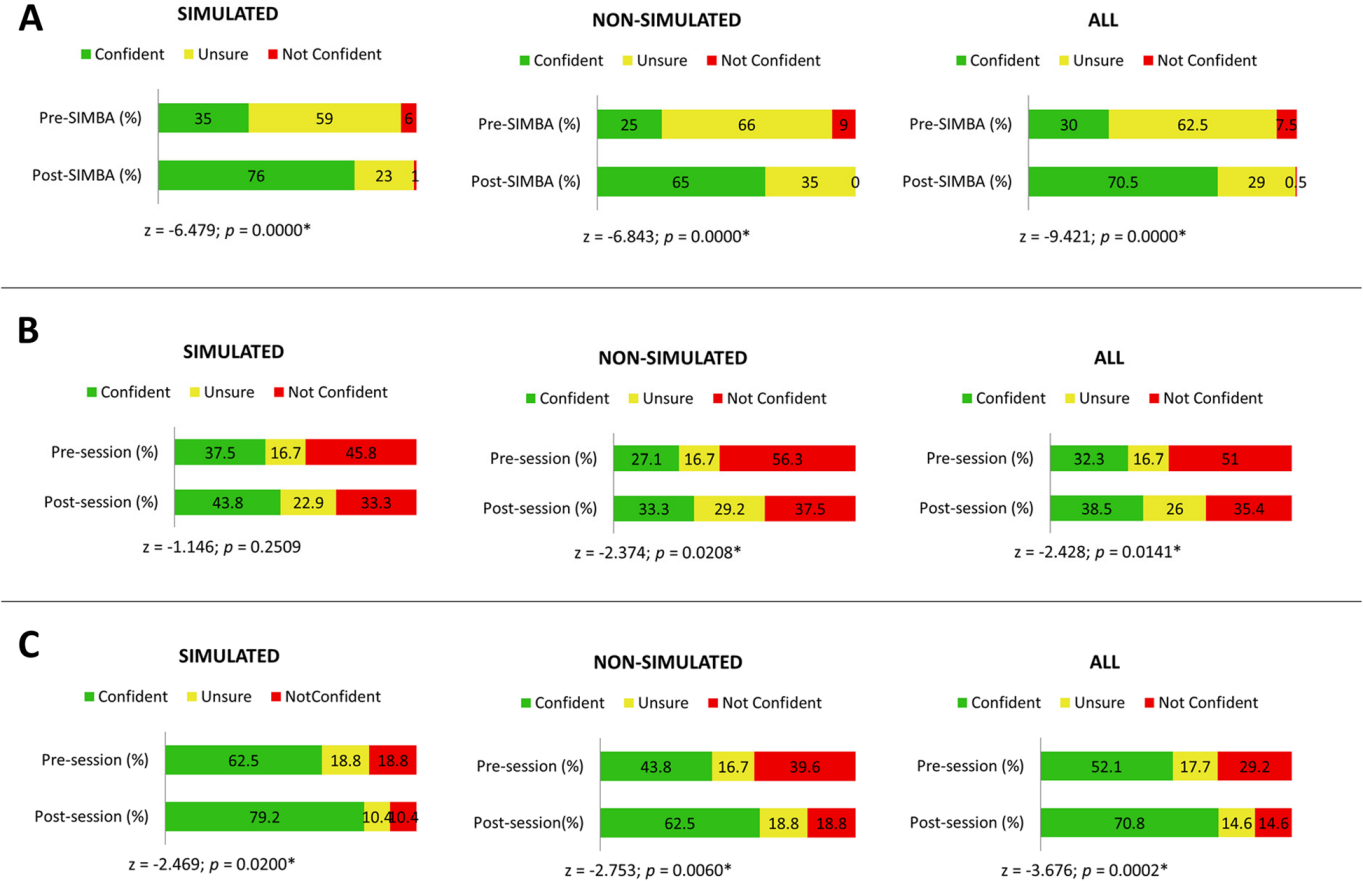
SIMBA-PCOS **A**) Illustration of changes in healthcare professionals’ confidence levels for managing simulated vs non-simulated cases. **B** Illustration of changes in confidence levels of women with PCOS for their confidence in HCPs to manage simulated vs non-simulated PCOS-related issues. **C** Illustration of changes in confidence levels of women with PCOS for their confidence in HCPs’ awareness of options available for managing simulated vs non-simulated PCOS-related issues

**Fig. 2:**
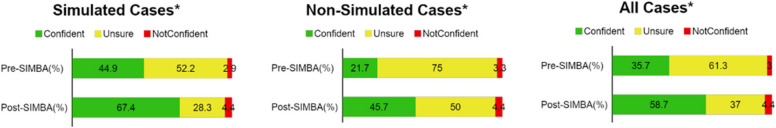
SIMBA-Adrenal conditions: An illustration of changes in healthcare professionals’ confidence levels for managing simulated vs non-simulated cases

**Fig. 3:**
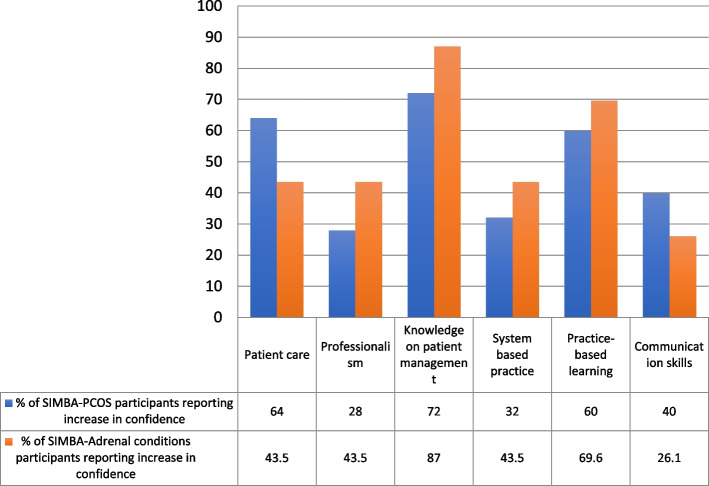
HCP’s Self-reported increase in confidence across competencies post-SIMBA-PCOS and SIMBA-Adrenal conditions

Statistical analysis of quantitative data was performed using STATA (STATA/SE 17.0 for Mac). Confidence levels are reported in frequencies and percentages and are presented as bar charts (Fig. 3). The Wilcoxon Signed-Rank test was used to compare pre- and post-SIMBA self-reported confidence levels. Statistical significance was accepted at a 95% confidence level. In addition, improvement in participants' confidence levels of simulated cases compared to non-simulated cases, followed by all sessions combined, was included in the comparison and reported as above (Figs. 1 and 2).

Post-SIMBA questionnaires for HCP also included open-ended questions regarding the overall feedback of the session. We performed a thematic inductive analysis for these open-ended questions to identify common feedback themes.

### Patient and public involvement statement

Various patient support groups and public were involved since the start of the project. Patients and public underwent workshop-style learning in parallel, using the same cases. HCPs, patients and public then came together to discuss the issues with the experts via Zoom. This promoted patient and public involvement in clinical decisions and care to achieve an equal partnership between healthcare professionals and patients alongside integrating PPI into medical education at both undergraduate and postgraduate levels. This model was codesigned by a PPI expert coordinator (CG).

## Results

### HCP Results

#### SIMBA-PCOS

Twenty-five out of 29 participants (86%) completed both pre- and post-SIMBA questionnaires. Characteristics of participants are described in Supplementary 7. Clinicians’ confidence in managing PCOS-related issues increased post-SIMBA for all eight domains of PCOS measured in this session (skin, weight, fertility, menstruation, menopause, diabetes, mental health, and endometrial cancer). Simulated cases and non-simulated cases showed a 41.0% (*p* < 0.001) and 40.0% (*p* < 0.001) increase in confidence, respectively, with all cases combined showing a 40.5% increase in confidence (*p* < 0.001) (Fig. 1A). There was an improvement in patient care (64.0%), communication skills (40.0%), professionalism (28.0%), knowledge of patient management (72.0%), system-based practice (32.0%), and practice-based learning (60.0%) (Fig. 2). 88.0% of SIMBA-PCOS participants strongly agreed/agreed that simulated topics applied to their practice, and 100% would attend similar SIMBA sessions in the future. Content analysis of feedback from SIMBA-PCOS participants to open-ended questions showed a positive rating and constructive comments for future sessions. Suggestions focused on three themes: time considerations, Q&A discussion format, and session content. Participants reflected on requiring more time for cases and discussion to allow for more questions to be answered. There was a call for more focus on assessing and managing the cases during the Q&A discussion.

#### SIMBA-Adrenal conditions

Twenty-three out of 27 (85%) HCP participants completed both pre- and post-SIMBA questionnaires. Characteristics of participants are described in Supplementary 7. Self-reported confidence increased post-SIMBA-Adrenal conditions for all ten adrenal conditions measured in the session (Addison’s disease, adrenal incidentaloma, adrenocortical carcinoma, Cushing’s syndrome, minimal autonomous cortisol secretion, primary aldosteronism, bilateral adrenal hyperplasia, congenital adrenal hyperplasia, phaeochromocytoma, and virilisation). Simulated cases and non-simulated adrenal cases showed a 22.5% (*p* < 0.001) and 24.0% (*p* < 0.001) increase in confidence, respectively, with all cases showing a 23.0% increase in confidence (*p* < 0.001) (Fig. 2). Participants reported improvement in confidence in practice-based learning (69.6%), system-based practice (43.5%), knowledge of patient management (87.0%), professionalism (43.5%), communication skills (26.1%), and patient care (43.5%) (Fig. 3). 95.6% strongly agreed/agreed that the simulated cases applied to their practice, and 100% would attend similar SIMBA sessions. Content analysis of feedback from SIMBA-Adrenal conditions participants to open-ended questions showed positive and constructive themes. Suggestions for improvement focussed on session length and structure, with participants reflecting on having smaller groups between chairs and participants for Q&A and using fewer cases to allow for a more in-depth discussion.

### Patient results

#### SIMBA-PCOS

Fifteen women with PCOS attended the session. Twelve (80%) completed both pre- and post-session surveys. Supplementary 8 shows the demographics of women with PCOS who participated in the session. The median age of PCOS diagnosis (*n* = 10) was 20.5 (IQR 19–25) years. 83.3% (*n* = 10/12) of people were aware of the criteria for diagnosing PCOS, and no significant difference after the session was noted (*p* = 1.000). People with PCOS reported their confidence in HCPs increased by 6.25% post-session (*p* = 0.0141) (Fig. 1B).

Similarly, people with PCOS’ confidence in HCPs’ awareness of options for managing PCOS-related issues increased by 17.7% (*p* = 0.0002) (Fig. 1C). 90% strongly agreed/agreed that SIMBA-PCOS benefit people with PCOS to better understand their condition and clinicians’ perspectives on the diagnosis and management of PCOS. In addition, 100% of people with PCOS strongly agreed/agreed that SIMBA-PCOS benefits clinicians in understanding patients’ perspectives on diagnosing and managing PCOS. Content analysis on open-ended questions was performed on all 12 participants who completed the post-SIMBA survey. Table 1 summarises these findings. Participants found the session informative, with engaging discussion and organised structure. Suggestions for improvement for future sessions centred around the format of the session advising breaking down the cases over sessions or reducing the number of cases to allow for future exploration of cases, together with organising structured feedback for future session discussions, allowing the equal contribution of all participants and representation from a broader range of patient groups.

**Table 1:** Themes identified from the workshop involving people with PCOS

Themes	Selected Quotes
Diagnosis experience of PCOS	“sort of like the mental health side of things, with a PCOS and that is diagnosis and university or, at any age is something which was neglected. My diagnosis and something which has been the most difficult part and having being somewhat of a history of an eating disorder which i'm very open about it was a massive massive trigger “
Clinicians seem to focus only on one symptom but not addressing other comorbidities	“But it's that patient having the knowledge to actually going through this treatment they need to look at the whole self that they're dealing with and that the fertility issues may then cause other issues and not be afraid to actually go and speak to the GP”
Adequacy of the information on PCOS given at diagnosis	“I just thought because she's so young she's at and it be helpful to explain that it's a lifelong condition and that the symptoms can be our spectrum and they look different in different people”
Patient centredness in the management of PCOS	“Knowing how long you're going to be on medication, and it is going to be successful, just explaining that there might be other options if it doesn't work could be something that might help with a bit of comfort, I think”

#### SIMBA-Adrenal conditions

Ten people diagnosed with adrenal conditions attended SIMBA-Adrenal conditions, and all 10 (100%) completed post-SIMBA surveys. Adrenal conditions represented were Addison's disease (*n* = 5), adrenocortical carcinoma (*n* = 3), Cushing’s disease (*n* = 1), and primary aldosteronism (*n* = 1). The median age of diagnosis with an adrenal condition was 39 (IQR 33–53) years. After the session, 70% knew the criteria for diagnosing their adrenal condition. 90% strongly agreed/agreed that SIMBA-Adrenal conditions benefited people with various adrenal conditions to understand their conditions better. 80% thought the session helped them understand clinicians’ perspectives on diagnosing and managing relevant adrenal conditions. Additionally, 80% of participants strongly agreed/agreed that SIMBA-Adrenal conditions benefit clinicians in understanding patients’ perspectives on diagnosing and managing adrenal conditions.

Content analysis was performed on all 10 participants’ feedback to open-ended questions. People with adrenal conditions revealed positive comments, including well-presented cases, open information sharing and a shared experience between doctor and patient (Table 2). Suggestions for improvement included bringing more doctors to discussion sessions having an agenda for discussion sessions alongside acknowledging individual circumstances and levels of understanding.

**Table 2:** Themes identified from the SIMBA-Adrenal conditions workshop

Themes	Selected Quotes
Seriousness of the disease	“When you have a crisis, you're that close to not being alive anymore. And I go from being conscious to unconscious in about five seconds”
Patients unsatisfied with the diagnostic process	“I had probably four years of a wasting disease that no one knew what the problem was”
Perceived lack of awareness from clinicians	“I was hospitalized with an Addison's crisis. … I had to explain to them what my problem was and what was going on”
Good experiences with healthcare professionals were seen as the exception	“Luckily one registrar at the hospital had seen it before and then they started doing all the tests hence the the diagnosis”
Patients taking management into their own hands	“One thing that I think could have been done better on discharge is to point her towards the self-help group. I think in particular with Addisons, but similar to diabetes, it's so important to talk to other patients because honestly the doctors do not know how to manage you. You have to find out yourself”
Suggested improvements to the case scenarios used in SIMBA session	“need to make sure we need to provide the participants about the u’s and e’s. So they are aware of that one too”

## Discussion

SIMBA-PPI sessions effectively improved clinicians’ confidence in managing PCOS and adrenal conditions. There was a greater improvement in HCP confidence in managing PCOS compared to adrenal conditions. This is likely due to a lower baseline confidence in managing simulated PCOS compared to adrenal conditions. For both SIMBA-PCOS and SIMBA-Adrenal conditions, most HCPs agreed that the simulated topics were applicable to their practice, and all participants indicated they would attend similar SIMBA sessions in the future. We acted upon HCPs’ feedback from the first SIMBA-PPI session, including allocating more time for case discussions and focussing more on assessment and management during the longer Q&A sessions. Feedback from HCPs for SIMBA-Adrenal was favourable for these changes. We will incorporate the suggestions for smaller groups for Q&A sessions and use fewer cases for more in-depth discussion in future sessions.

People with PCOS and adrenal conditions agreed that SIMBA-PPI sessions helped them learn more about their needs and understand clinicians’ perspectives on diagnosis and management. Our findings suggest there is a gap in knowledge and expectations between patients and HCPs involved in their care, and this gap can be reduced by attending SIMBA sessions involving both affected people and HCPs.

Drawing from theories such as Experiential Learning [20] and Participatory Action Research [10], the SIMBA-PPI model places patients and the public at the forefront. By recognising the importance of community engagement and empowerment, Community-Based Participatory Research [32] (CBPR) principles are woven into the fabric of the SIMBA-PPI model, emphasising collaboration, relevance, and cultural responsiveness. This educational framework aligns with the Health Belief Model [11] and Theory of Planned Behaviour [12, 13], acknowledging the significance of individual beliefs, attitudes, and readiness to engage in healthcare decisions. At its core, the SIMBA model harnesses the power of Empowerment Theory [13] and Social Cognitive Theory [14] to cultivate an educational environment that educates future healthcare providers and empowers patients and the public to participate in their care actively. It champions the development of a new generation of healthcare professionals who value partnership, empathetic communication, and collaboration with patients and communities.

To our knowledge, this is the first educational activity with PPI to identify the knowledge gap between HCPs and people with PCOS and adrenal conditions. The efficacy of SIMBA as an educational tool in improving clinicians’ confidence in managing simulated cases, with a high acceptance rate, is consistent with our previous SIMBA sessions on diabetes, endocrinology and acute medicine [16, 18, 33, 34]. In SIMBA, participants completed three stages of Kolb’s experiential learning cycle before going into the fourth stage—active experimenting -, when they can apply the knowledge learnt from SIMBA into their real-life clinical practice. In SIMBA-PPI, the patients provided additional learning to the HCP participants at each of the three stages of the learning cycle. The experience shared by the patients enabled HCPs to reflectively observe and form a better conceptualisation of the content through lived experiences.

Several studies have highlighted the gap in knowledge, attitude, and perception of clinical care between HCPs and people with PCOS and adrenal conditions. Delays in diagnoses of PCOS and inadequate information given to people with PCOS upon diagnosis have been reported worldwide [23, 24, 35]. Poor experiences with diagnoses and management of PCOS have been associated with increased anxiety and depression, further negatively impacting self-management [36–38]. This has led to patients and their carers relying on mostly non-evidence-based information online, potentially containing inaccuracies and contradicting what patients have been advised by the HCPs [36]. Similarly, patients with adrenal conditions often receive late diagnoses [39–41]. Previous studies have also shown that patients were unaware of appropriately adjusting steroid doses during acute illness due to suboptimal and inconsistent education [40, 41]. SIMBA-PPI helps to address this issue by bringing HCPs and patients together to share real-life experiences and provides a platform where both can teach and learn from each other [36, 39]. Such educational events can also help patients understand evidence-based strategies and clinicians' limitations. This may potentially reduce any unrealistic expectations of the patients and the gap in knowledge and expectations between patients and HCPs.

The content analysis of feedback from open-ended questions identified several areas for improvement as part of the PDSA cycle. Further feedback from SIMBA-Adrenal conditions suggests future sessions should be more tailored to individual patient circumstances and different levels of understanding. Additional work is needed to determine whether our findings of increased self-reported confidence levels translate to a long-term improvement in clinical performance and patient-reported outcome measures.

The integrative SIMBA model envisions a curriculum where students learn from, with, and about patients and the public. Through immersive, experiential learning opportunities, students engage in authentic healthcare scenarios, fostering critical reflection, and applying insights gained from collaborative interactions. It promotes the creation of inclusive spaces where patients, healthcare professionals, educators, and researchers co-create knowledge and solutions, thereby bridging the gap between theory and practice. In essence, the SIMBA patient public involvement medical education model represents a transformative approach, transcending traditional pedagogical boundaries to nurture a healthcare ecosystem rooted in empathy, collaboration, and patient-centred care.

While the study has several strengths in the design and outcomes, we acknowledge that some factors may limit our findings' generalisability, one being that the results are from only two sessions. Our PPI participants were invited through various support groups and were interviewed for their interest in participating. This may be considered as a potential selection bias due to this convenience sampling method. However, the interview was necessary to ensure their engagement for the entire duration of our session. Another limitation was that for SIMBA-Adrenal conditions due to the split between the workshop and the HCP simulation, many PPI participants did not attend the discussion session; hence, post-SIMBA questionnaires were not filled. This can be mitigated in the future by reducing the time gap between the workshop and HCP simulation, ensuring clear communication, sending timely reminders and engaging participants actively in the process. In addition, our small sample size may also impact our results and may not be representative of the general patient population. However, applying a mixed-method approach helped consolidate the findings from the study. The study questionnaires were not validated, which could affect their reliability.

Follow-up studies based on the principles of Diffusion of Innovation Theory [42] and Transtheoretical Model [43] are needed to study long-term impact. Further studies will look into the differences in opinions, experiences, and expectations of HCPs trained in the UK versus abroad. Similarly, we want to incorporate a wider perspective of patients from different backgrounds. Following the successful implementation of SIMBA in undergraduate education, we are looking to introduce PPI. We also want to implement SIMBA-PPI as a continuing professional development activity for specialist trainees.

## Conclusion

SIMBA-PPI is an effective learning tool for people with PCOS and adrenal conditions and their healthcare professionals to enhance their understanding of the condition. SIMBA is open access and cost-effective. The model also helped identify and reduce gaps in knowledge and expectations between HCPs and patients with PCOS and adrenal conditions. Future large-scale studies are needed to study the long-term impact on clinical practice and patient-reported outcome measures.

### Supplementary Information

Supplementary Material 1.

## Data Availability

Data available on request to the corresponding author.
