# Supplementary material for: Reducing the Gap in Knowledge and Expectations between Clinicians and People with Polycystic Ovary Syndrome or Adrenal Conditions: Simulation via Instant Messaging—Birmingham Advance: Patient and Public Involvement (SIMBA-PPI) Study
Source: BMC Med Educ. 2024 Jul 22;24:784. doi: 10.1186/s12909-024-05772-w (PMC11265159; doi:10.1186/s12909-024-05772-w)
Supplement: Supplementary file 1 — Supplementary Material 1. [file 12909_2024_5772_MOESM1_ESM.docx]

**Supplementary 1.** Brief explanations of educational theories and frameworks.

| Educational theory/framework | Citation | Brief explanation |
| --- | --- | --- |
| Participatory action research | 10 | A collective, self-reflective investigation conducted by researchers and participants to understand and enhance the practices and situations they are involved in. |
| The Health Belief Models | 11 | A psychological framework that predicts health behaviours based on individuals' perceptions of the threat posed by a health problem, the benefits of avoiding the threat, and the barriers to taking preventive action. |
| The theory of planned behaviour | 12 | A psychological theory that predicts an individual's intentions and behaviours based on their attitudes, subjective norms, and perceived control over the behaviour. |
| Empowerment theory | 13 | A psychological theory that focuses on enabling individuals and communities to gain control, improve their lives, and achieve their goals through the development of skills, confidence, and access to resources. |
| Social cognitive theory | 14 | Learning occurs in a social context and can result from observation, imitation, and modelling, influenced by the interplay between personal factors, behaviours, and environmental influences. |
| Simulation game | 19 | A structured, interactive experience that replicates real-world processes and allows participants to make decisions and observe their consequences within a controlled environment. |
| Kolb’s experiential learning theory | 20 | Effective learning is a four-stage cyclic process consisting of concrete experience, reflective observation, abstract conceptualisation and active experimentation. |
| Community-based participatory research | 33 | A collaborative research approach that inclusively engages community members, researchers, and stakeholders in the research process, acknowledging and leveraging the unique strengths each participant contributes. |
| Diffusion of innovation theory | 45 | Explains how new ideas, products, or practices spread through society over time, influenced by the characteristics of the innovation, adopters, communication channels, and social context. |
| Transtheoretical model | 46 | A psychological theory that describes the stages individuals go through when changing behaviour, emphasizing processes and strategies to facilitate successful behaviour change. |

**Supplementary 2**. The plan-do-study-act model adopted for our study.


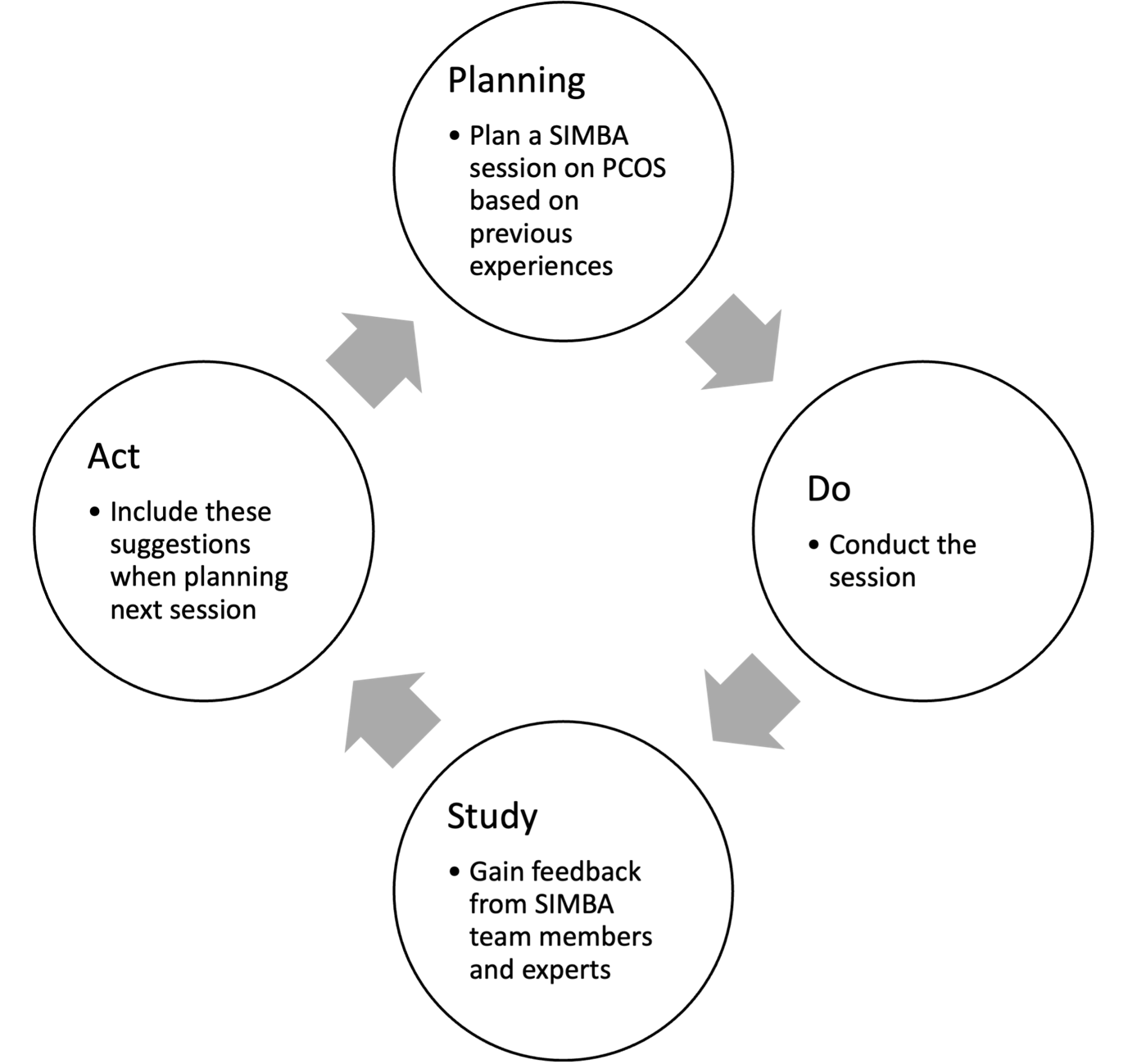


**Supplementary 3A.** Overview of SIMBA-PCOS session.


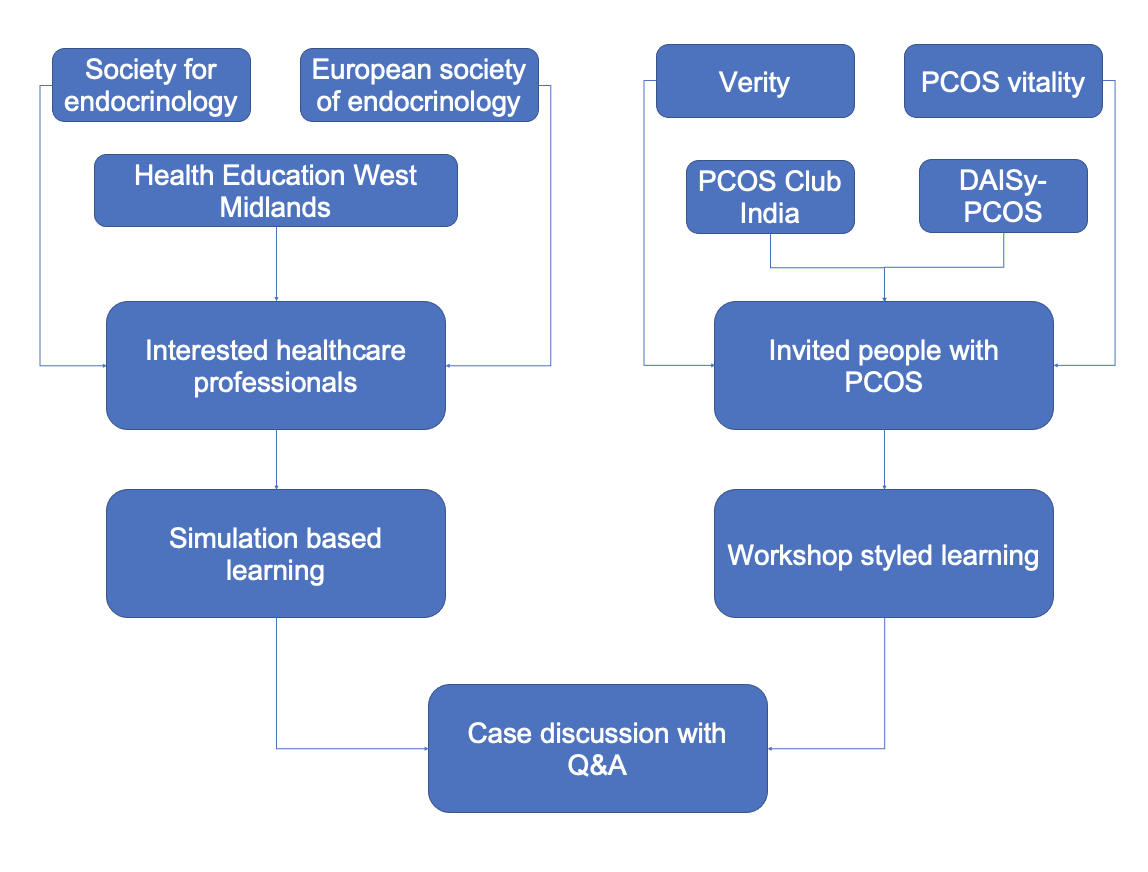


**Supplementary 3B.** Overview of SIMBA-Adrenal session


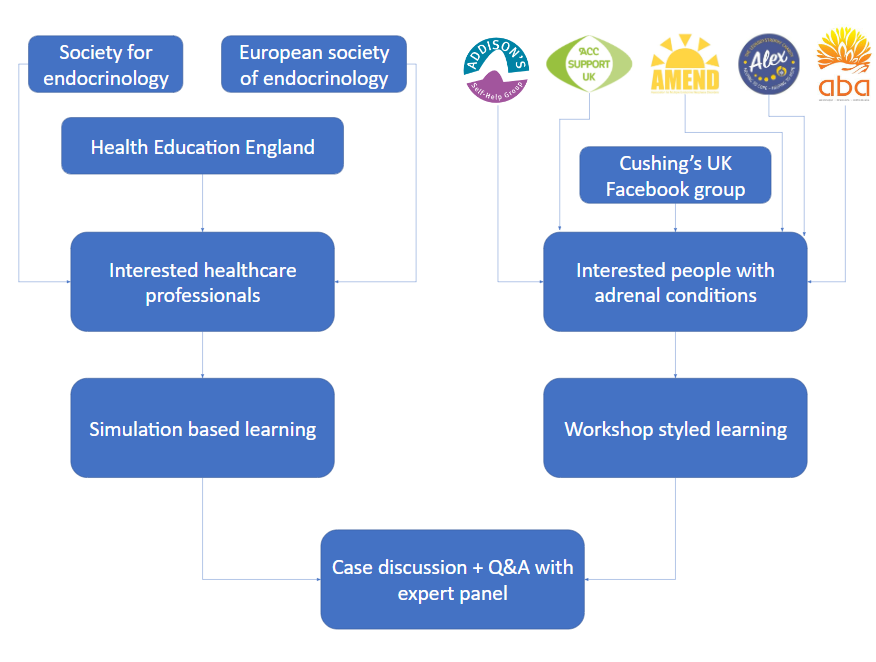


**Supplementary 4A.** Example of SIMBA PCOS transcript.

SIMBA PCOS Case 4 - Skin

**Instruction to moderators:** If information asked by participants is not available in the transcript, please copy the following

*The information you requested is not available.*

**Please liaise with your senior moderator if any doubts at any point of the simulation**

**Instruction to moderators:** Copy the following before the simulation starts.


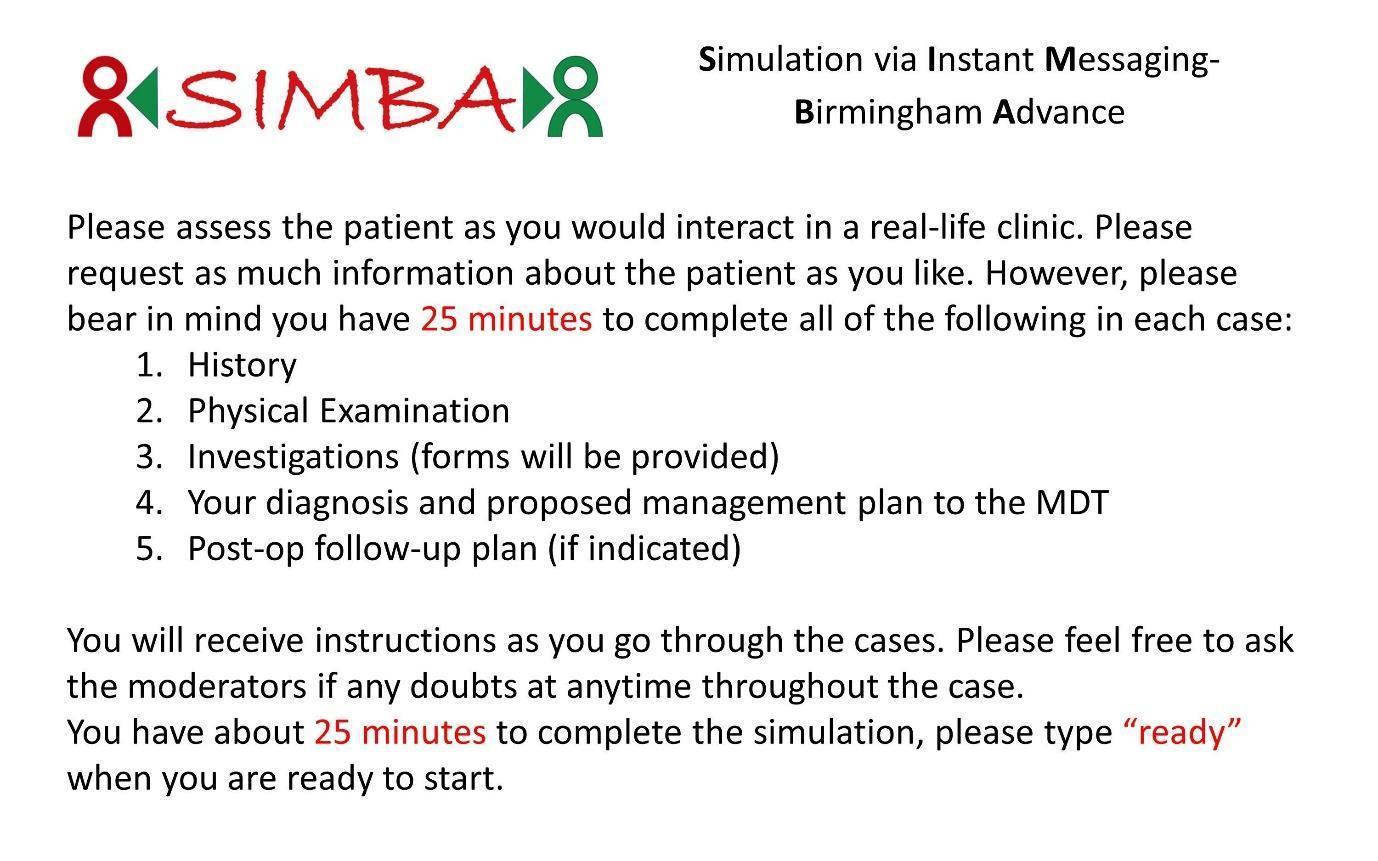


**Instruction to moderators** After participants are ready, copy the presenting complaint below

Presenting complaint

A 24-year-old female presented to General Practice with absent menstrual periods.

Please assess this patient and request for any information you will need in your assessment.

# **History of presenting complaint**

After stopping my oral contraceptive pill six months ago, I noticed absent menstrual periods. Since then, I have also significantly gained weight. I have gained around 5-7kg over 12 months.

My menstrual cycle was previously regular. I am otherwise well.

Past medical history and surgical history

- Asthma
- Increased BMI (42 kg/m2)

# **Medications**

- Salbutamol
- Combined oral contraceptive pill - stopped 6 months ago (Ethinylestradiol 20mcg/ levonorgestrel 100mcg).

# **Allergies**

I do not have any allergies.

# **Family history**

I do not have a family history of conditions.

# **Social history**

I smoke five cigarettes every day and occasionally drink alcohol.

**Instructions to moderators:** After the participants have asked all the information above please copy the following:

You have now completed history taking. What examinations would you like to perform for this patient?

Examinations: **Ensure to give only the requested section for examinations**

*Observations:*  BMI 42kg/m2; BP 140/85

*Systemic examination:* The cardio-respiratory exam was unremarkable.

*Speciality specific examination:* An abdominal exam was performed to look for striae or other features of hypercortisolism, including assessment for proximal muscle weakness. Axillae and neck were inspected for skin tags or acanthosis. No abnormality was detected, and gynaecological exam was also normal.

**Instruction to moderators** After the participants have asked all the information above please send them the blood form and copy the following.

You have gathered all the required information.

Please follow the link provided, tick all the relevant investigations you need for the case, and *text the moderator after you have submitted the form.*

<https://forms.gle/3pkwk7FbjqMzLojf8>

Results will be provided.

**Instructions to moderators:** send all the investigations as images from below (blood, dynamic function test) **after you have confirmed that they submitted the investigations form**.


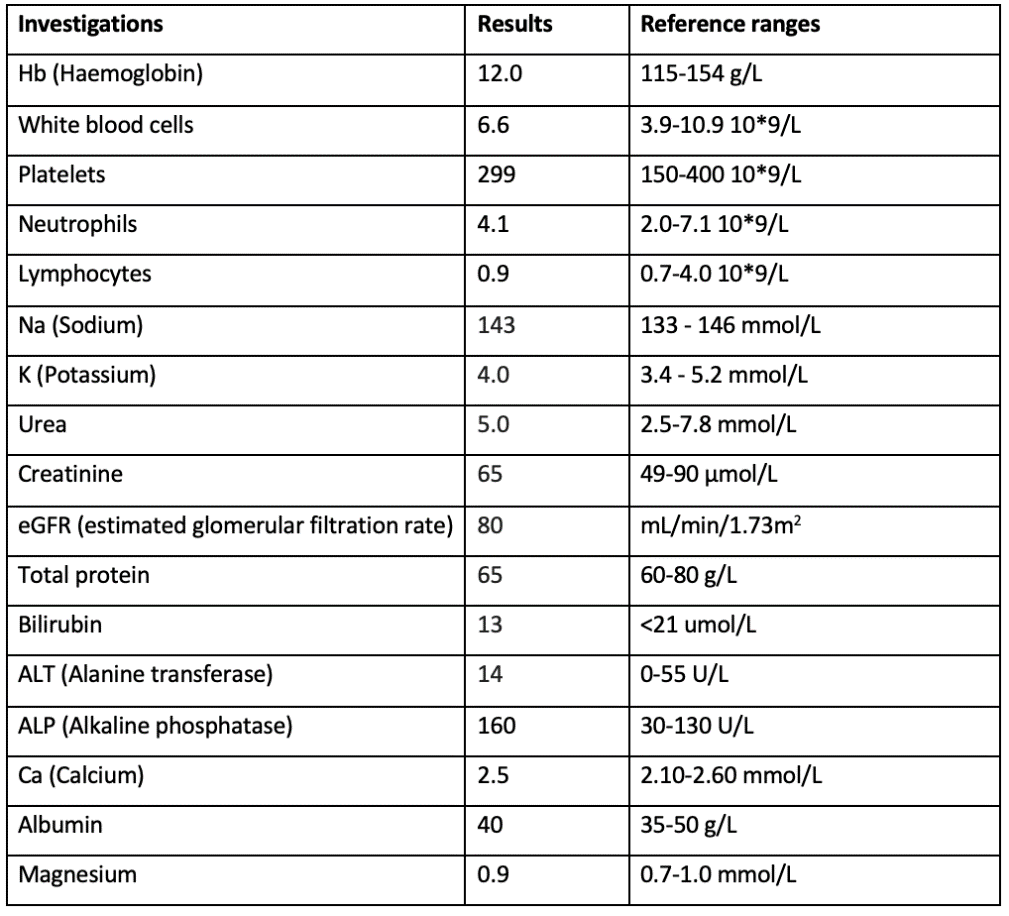


*Endocrinology serum tests*:


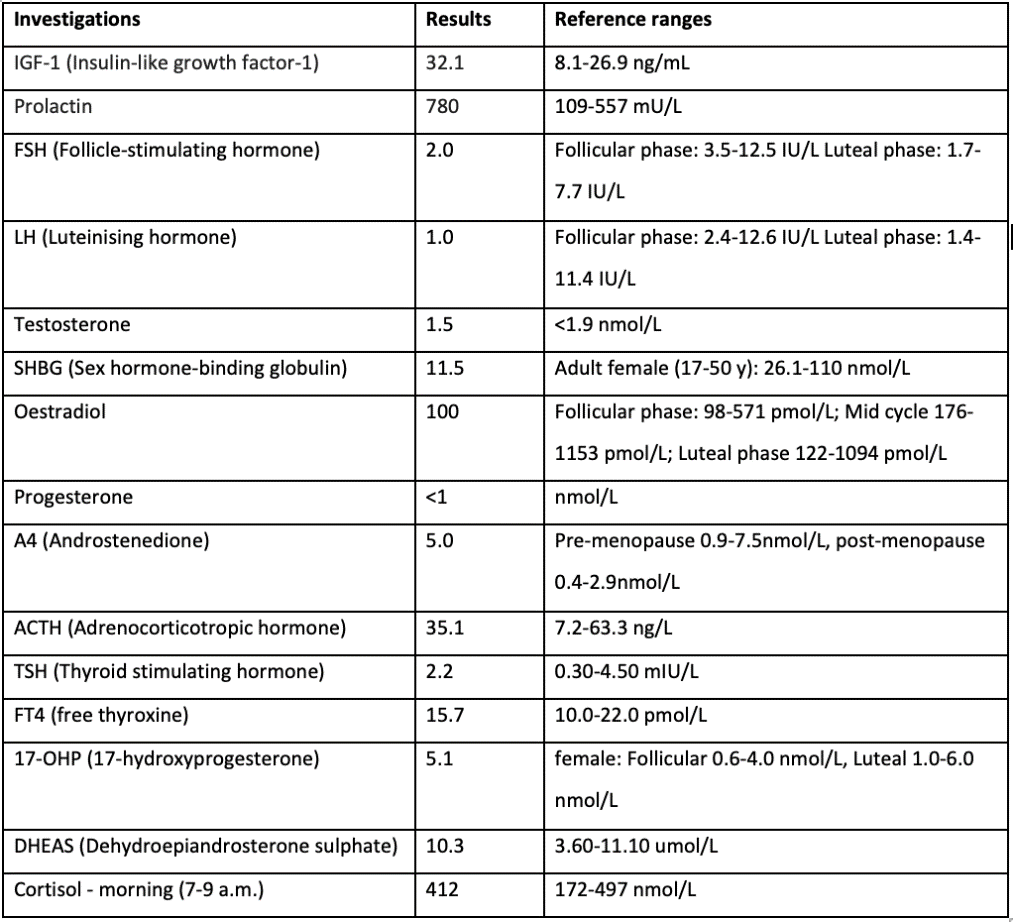


**Instructions to moderators:** If the participant asks for imaging, please send the following:

Please follow the link provided, tick all the relevant imaging studies you need for the case, and *text the moderator after you have submitted the form.*

<https://forms.gle/uwkqbieWkrGTwq418>

Results will be provided.

**Instructions to moderators:** **After you have confirmed that they submitted the investigations form**, copy the following:

The MRI pituitary shows no abnormality.

The transvaginal pelvic ultrasound showed bilateral polycystic ovaries (PCO). Ovarian volume 10-12 ml bilaterally with multiple peripheral follicles in typical PCO distribution.

**Instructions to moderators:** After the participants have asked all the information above, please copy the following:

You have now gathered all the available information for this case.

Please write to the MDT:

- state the differential diagnosis (and its rationale)

- propose management and follow-up/discharge plan (if any)

**Instructions to moderators:** please copy below after the trainee has answered above questions.

*MDT outcome:*

The differential diagnosis includes polycystic ovarian syndrome (PCOS), post-pill amenorrhoea, obesity-related amenorrhoea, Cushing’s syndrome, and acromegaly.

A progesterone withdrawal challenge was performed. The patient had a menstrual response to medroxyprogesterone acetate. Cortisol excess was excluded, and a hysteroscopy was also performed. The mainstay of treatment included weight loss and metformin. The patient was later referred for bariatric surgery as well.

**Instructions to moderators:** After you have sent the text above, please copy the following:

*The simulation has ended. Many thanks and we will discuss the cases shortly.*

**Supplementary 4B.** Example of SIMBA Adrenal transcript.

SIMBA Adrenal Conditions Case 4 – Cushing’s Disease

**Instruction to moderators:** If information asked by participants is not available in the transcript, please copy the following

*The information you requested is not available.*

**Please liaise with your senior moderator if any doubts at any point of the simulation**

**Instruction to moderators:** Copy the following before the simulation starts.


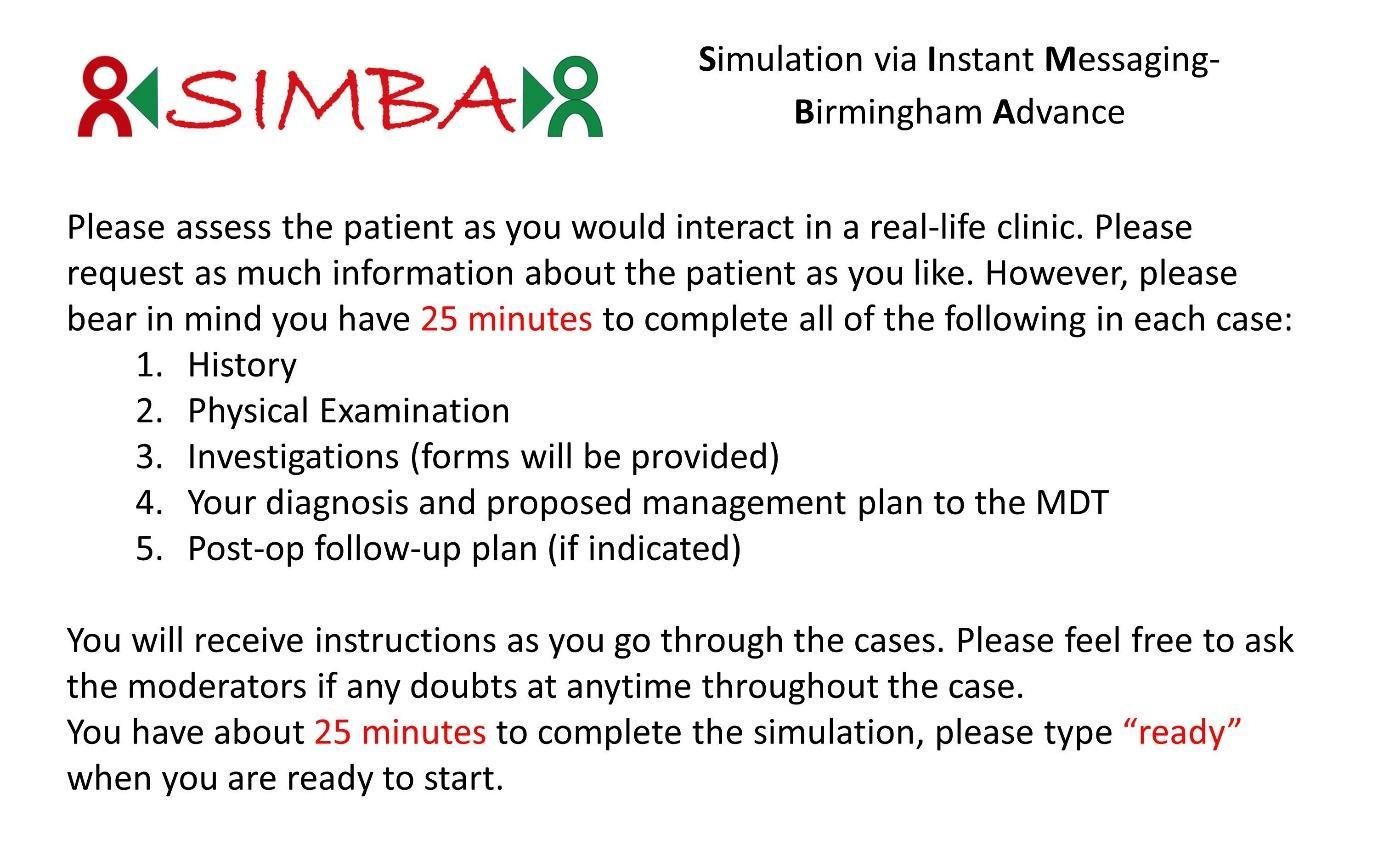


**Instruction to moderators** After participants are ready, copy the presenting complaint below

Presenting complaint

A 74 year old woman was referred to the endocrine clinic due to an adrenal incidentaloma on CT scan.

Please assess this patient and request for any information you will need in your assessment.

# **History of presenting complaint**

I have noticed that I have been gaining a lot of weight particularly around my waist or belly area. In the past two years, I have gained about 20 kg. Here are the scans:


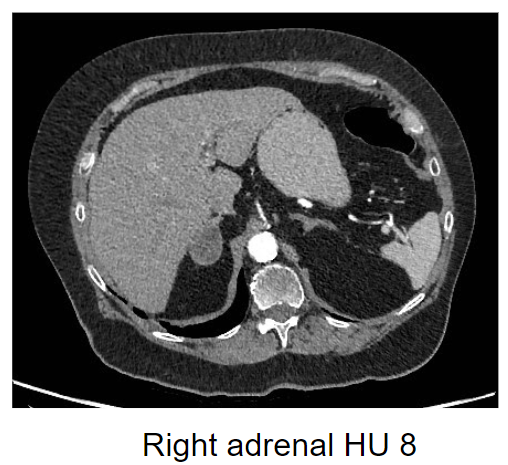


High resolution CT scan of thorax, abdomen and pelvis

Past medical history and surgical history

I have had hypertension for 20 years and osteoporosis.

# **Medications**

At the moment I am on:

1. Perindopril
2. Atenolol
3. Doxazosin
4. Simvastatin
5. Alendronic acid
6. Cholecalciferol

# **Allergies**

I don’t have any allergies.

# **Family history**

No significant family history.

# **Social history**

No significant social history.

**Instructions to moderators:** After the participants have asked all the information above please copy the following:

You have now completed history taking. What examinations would you like to perform for this patient?

Examinations: **Ensure to give only the requested section for examinations**

*Observations:* 157cm. 81.6kg. BP 180/95

*Systemic examination:* Skin thin ++. Facial plethora ++. Some bruising

*Speciality specific examination:* Leg exam: proximal myopathy ++

**Instruction to moderators** After the participants have asked all the information above please send them the blood form and copy the following.

You have gathered all the required information.

Please follow the link provided, tick all the relevant investigations you need for the case, and *text the moderator after you have submitted the form.*

<https://docs.google.com/forms/d/e/1FAIpQLSca1u-7vmW_CER09eSccIBPic7ipHs5JkjLAUXL8drmBg8aZA/viewform?usp=pp_url>

Results will be provided.

**Instructions to moderators:** send all the investigations as images from below (blood, dynamic function test) **after you have confirmed that they submitted the investigations form**.


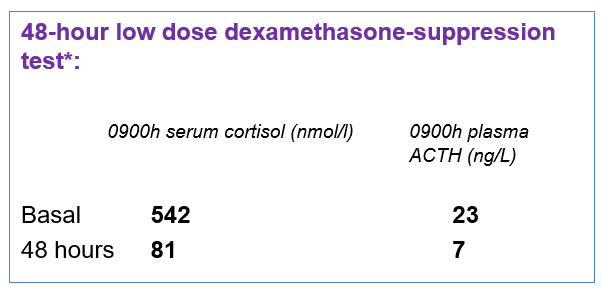


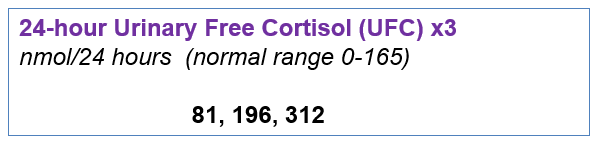


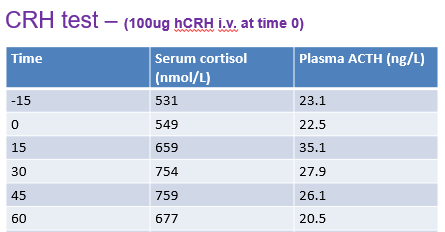


**Instructions to moderators:** If the participant asks for imaging, please send the following:

Please follow the link provided, tick all the relevant imaging studies you need for the case, and *text the moderator after you have submitted the form.*

<https://docs.google.com/forms/d/e/1FAIpQLSc9niKHhvVmX0Cr0VJFkqYj_j5jGzCyjy-TZI-PR3E4DF5dkA/viewform?usp=pp_url>

Results will be provided.

**Instructions to moderators:** send images from below **after you have confirmed that they submitted the investigations form** and copy the following.

Please interpret the imaging findings.

Pituitary imaging:

Gadolinium T1:


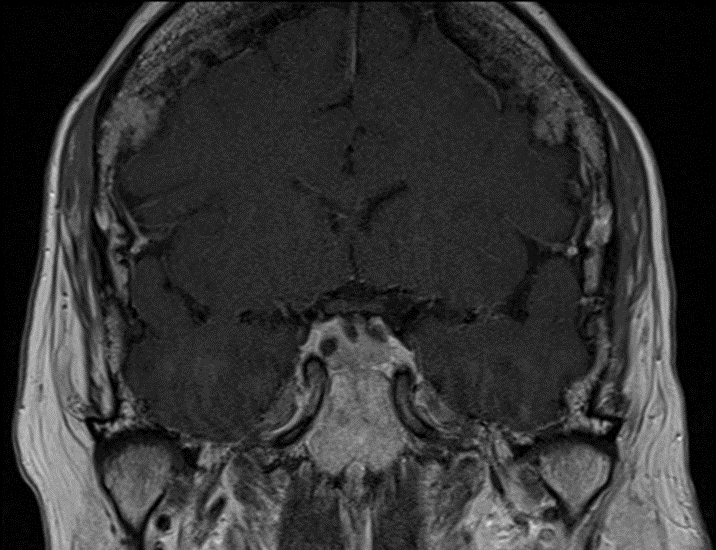


T2:
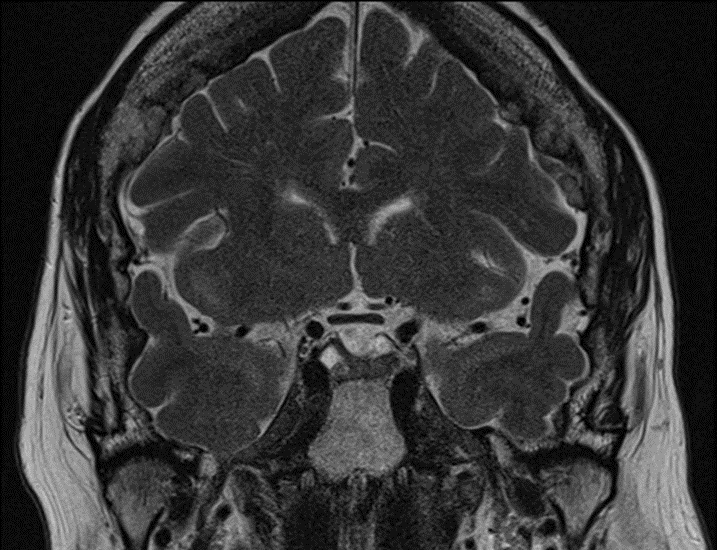


**Instructions to moderators:** send images from below **after you have confirmed that they submitted the investigations form** and copy the following.

Please interpret the imaging findings.

**Instructions to moderators:** after the participant has interpreted the image, please send the following

- Normal CT head.
- Right adrenal incidentaloma – remaining adrenal limbs hyperplastic
- Adrenal mass most likely non-functioning (but autonomy possible)

**Instructions to moderators:** After the participants have asked all the information above, please copy the following:

You have now gathered all the available information for this case.

Please write to the MDT:

- State the differential diagnosis (and its rationale)

- Propose management and follow-up/discharge plan (if any)

**Instructions to moderators:** please copy below after the trainee has answered above questions.

Cushing’s disease – clinically and biochemically

Management plan:

1. Pituitary surgery discussed with patient (transsphenoidal surgery)
2. Possibility of autonomous adrenal discussed with patient
3. Warned that she would likely feel worse for 6-18 months after surgery if successful

**Instructions to moderators:** please send the following as a separate message.

The histology of the patient returned with the following:

- Corticotroph adenoma ACTH + immunoreactivity
- Ki67 index – low
- Crooke’s hyaline change in non-neoplastic adenohypophysis

*How would you manage this and what would be your plan for follow-up?*

**Instructions to moderators:** please copy below after the trainee has answered above questions.

Pre-morning hydrocortisone (HC) 0900h serum cortisol (nmol/L) (initially HC 10/10/5 reduced to 10/5 by 6 months post-op)


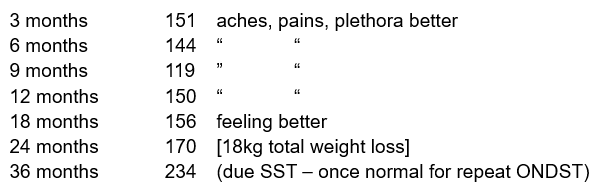


**Instructions to moderators:** please send the following as a separate message.

*The simulation has ended. Many thanks and we will discuss the case shortly.*

**Supplementary 5.** Previously published detailed description of the various steps involved in the SIMBA session. Taken from: *Melson, E. et al. Simulation via instant messaging-Birmingham advance (SIMBA) model helped improve clinicians’ confidence to manage cases in diabetes and endocrinology. BMC Med Educ 20, 1–10 (2020).*

We initially identified five real-life case scenarios for endocrine and diabetes sessions. Following approval from specialists, anonymised transcripts were created on these case scenarios. These transcripts included medical history, clinical examinations, investigation results, imaging and other relevant information that would enable trainees to diagnose the case, propose management and follow-up plans. No patient identifiable data was included in the transcript. These transcripts were validated and approved by a consultant endocrinologist with specialist expertise to ensure that they portray real-life scenarios of respective cases. While the images used in endocrine session were approved by a consultant neuro-radiologist with a special interest in pituitary pathology, similar approval was obtained for continuous glucose monitoring and Libre readings for diabetes session (Fig. 1).


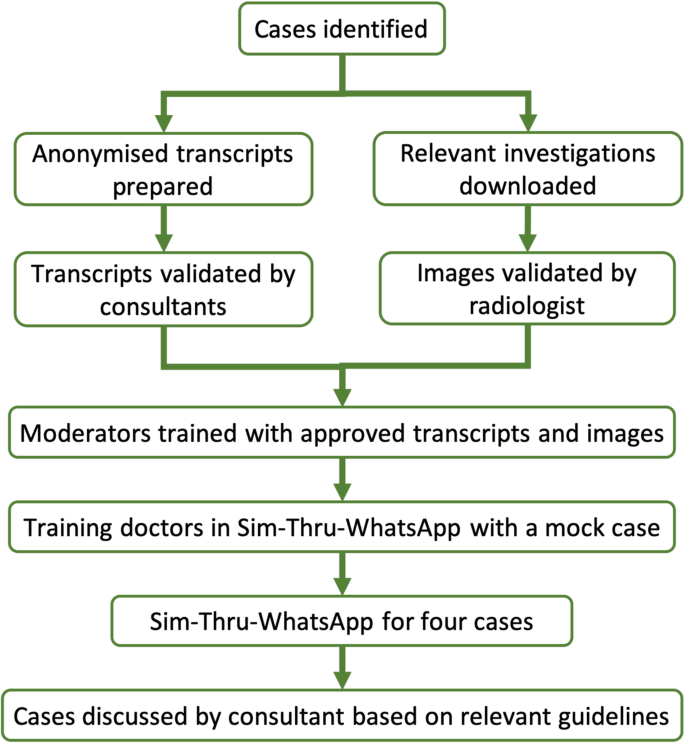


**Fig.1** SIMBA protocol

For the endocrine session, standardized transcripts of five anonymized pituitary cases — Non-Functioning Pituitary Adenoma (NFPA), craniopharyngioma, macroprolactinoma, acromegaly and Cushing’s disease — were prepared. For diabetes session, standardized transcripts of four anonymized diabetes cases — interpreting Libre readings, interpreting continuous glucose monitor (CGM) readings, hypoglycaemic unawareness, and monogenic diabetes — were created. Along with the simulated case scenarios, non-simulated case scenarios were identified by the consultant specialist supervising the specific session based on their prevalence and relevance to daily clinical practice. Non-simulated cases were different from simulated scenarios but matched in frequency, exposure and challenge to simulated ones in real-world practice for participants. This was to match simulated and non-simulated scenarios as much as possible so that SIMBA was the only variant between the two. The idea behind the analysis comparing simulated and non-simulated cases was for the participant to draw their experience from day-to-day practices while reporting their confidence to manage such case pre- and post-SIMBA session. For example, in diabetes session, Libre and CGM were compared with blood glucose meters and ketone meters; hypoglycaemic unawareness was compared with neuropathy and monogenic diabetes was compared with gestational diabetes.

In the endocrine session, five moderators were chosen to participate in the study, and the number of moderators was increased to ten for diabetes session based on the feedback from the endocrine session. The moderators were recruited considering their interest in the field of endocrinology and their motivation to participate in the innovative method of learning. To ensure their proficiency, the selected moderators were provided with the finalized transcripts 3 weeks prior to the session. The moderators familiarized themselves with the transcripts followed by at least five mock simulation sessions amongst each other. In order to ensure there was no heterogeneity in the responses, these moderators were then tested by the senior authors of the study. The role of the moderators was to simulate a patient, a senior clinician and a multi-disciplinary team (MDT) liaison at different points of the simulation. At the start of the simulation, the moderator took up the role of a patient from whom trainees requested a history of presenting complaints and relevant associated medical history. Where the trainees requested physical, biochemical, radiological or any other relevant examination results, moderators simulated a senior clinician to provide this information. Lastly, the moderators played the role of MDT liaison when the trainee combined all relevant information to arrive at the diagnosis, management and follow-up plans. Moderators were instructed to give the relevant information that is provided in the transcripts. If they were asked for information not provided in the transcript, they would reply saying “this information is not available”.

On the day of the simulation, each moderator was assigned to a small group of trainees (two to four) with whom they would be replying in parallel. All trainees had been asked in advance to bring their own computers/laptops/notebooks through which they connected with the moderators via WhatsApp® Web application. The session started with the information in Fig. 2 via WhatsApp®. In summary, the trainees were instructed to approach the cases as they would in their daily clinical practice. Once the trainees were ready, the simulation was initiated by providing them with the presenting complaint of the patient. At this point, the moderator played the role of a patient answering relevant questions from the trainees to provide medical history. Once the history taking was complete, the moderators took up the role of a senior clinician providing necessary and relevant examination findings when asked by the trainee. The moderator prompted the trainee when they completed history and examination advising them to move to the next step of evaluation. If the trainee then proceeded to request blood tests, they were sent an electronic blood tests form to request the necessary investigations. Once the completed form was returned via WhatsApp, the moderators replied with the results of the requested blood test. If the trainee proceeded for a dynamic function test and/or radiologic investigation, the process for request and provision of results as for blood investigations was repeated. Once these steps were complete, the moderator prompted the trainee to summarise the findings and propose the diagnosis and management plan to MDT. As these cases had been discussed in MDT in real-life, the moderator compared the trainee’s reply to the factual outcome. Should they match, the moderator informed the same to the trainee. If they did not match, the moderator advised the trainee with the correct diagnosis. In both scenarios, the moderator asked the trainee for a follow-up plan. Once the trainees provided follow-up plans, the simulation ended. If at any point during simulation, a trainee requested information that was unavailable on the transcript (e.g. ordering an inappropriate investigation or skipping a crucial step in diagnosis/management), they were prompted by the moderators that the information was not available or was appropriately guided back to the relevant step.


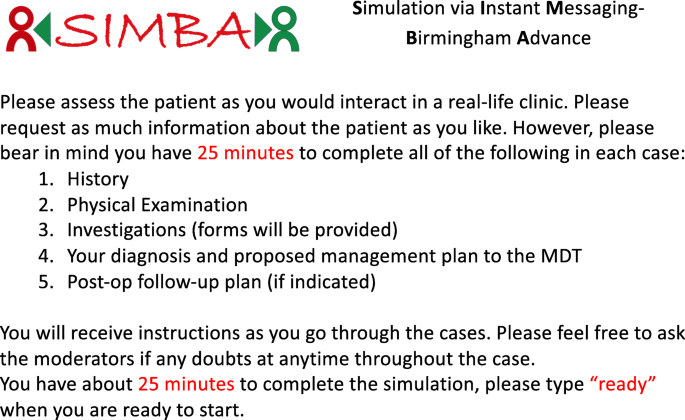


**Fig.2** Instructions provided pre-simulation

To allow participants to familiarize themselves to the simulation model, the first case in each session was run as a trial. After the first case, the case and its approach were discussed in detail in line with current guidelines by an expert, which further helped the trainees to better understand the course of the simulation session. We include an example WhatsApp transcript as a supplement to the paper for a better understanding of the model.

During the endocrine session, the case scenario of non-functioning pituitary adenoma (NFPA) was run as a trial. Following this, the trainees underwent simulation case scenarios for macroprolactinoma, craniopharyngioma, acromegaly and Cushing’s disease, followed by respective case discussions with consultant endocrinologist.

During the diabetes session, the case scenario of interpreting Libre readings was chosen as a trial similar to NFPA in endocrine session. This was followed by case scenarios for interpreting CGM readings, hypoglycaemic unawareness, and monogenic diabetes, followed by case discussions with relevant approaches in detail.

During post-simulation discussions, the consultant focused on the appropriate approach to the cases, in relation to the evidence-based international, national and local hospital guidelines as appropriate, in that order of hierarchy, for each specific condition. Structured feedback/debriefing occurred at the end of the session when the chair discussed the cases and highlighted the lessons learned by getting the trainees to reflect on their performance and discuss strategies for using these lessons to improve their daily practice. The discussions were interactive, and the trainees had ample opportunity to ask any further questions regarding the diagnosis or management of the simulated cases. The trainees were not ranked or scored on their performance. However, they were informed about the accuracy of their diagnosis during the simulations by the moderators as described above.

The confidence of the trainees (measured using a Likert scale ranging from strongly disagree to strongly agree) in approaching different pituitary and diabetes cases was assessed pre- and post-simulation [27, 28]. These data were then categorised into three groups: (i) confident: for those who responded with strongly agree and agree; (ii) not confident: for those who responded with disagree and strongly disagree; (iii) unsure: for those who responded with agree somewhat, disagree somewhat and undecided. The confidence levels of managing cases pre- and post-simulation are reported using frequencies, percentages, and are displayed in bar charts. Due to the nature of the data, Wilcoxon rank-sum tests (significance set at p < 0.05) were deemed appropriate (using STATA MP/4 (Statacorp 2017)) to statistically compare confidence levels pre- and post-simulation. Significant tests are highlighted using an asterisk.

Improvements in trainees’ confidence levels pre- and post-simulation of simulated scenarios (endocrine session – NFPA, craniopharyngioma, macroprolactinoma, acromegaly, Cushing’s disease; diabetes session - interpreting Libre reading, interpreting CGM reading, hypoglycaemic unawareness, and monogenic diabetes) vs. non-simulated scenarios (endocrine session – microprolactinoma, pituitary apoplexy, thyrotropinoma, gonadotropinoma, pituitary carcinoma; diabetes session - neuropathy, gestational diabetes, blood glucose meters, and ketone meters) were also displayed using frequencies, percentages, bar charts, and were also statistically tested using Wilcoxon rank-sum.

In addition to views on the management of the cases, trainees were also asked to comment on their overall impression of the session, the consultant’s contribution during the discussion and their interaction with the moderators.

**Supplementary 6A.** Example of Pre-SIMBA-PPI survey for patients.

**Pre-SIMBA-PCOS survey (Patients)**

I consent voluntarily to be a participant in this study and understand that I can
refuse to answer questions and I can withdraw from the study at any time,
without having to give a reason, up until the online survey is submitted; and that

after the survey is submitted it will not be possible to withdraw (as it will not be

possible to link you personally with the information submitted online).

(Yes/No)

Diagnosis

I am satisfied with my diagnosis experience of PCOS- strongly agree, agree, neither agree or disagree, disagree, strongly disagree

My clinician(s) provided myself with adequate information on PCOS at diagnosis- strongly agree, agree, neither agree or disagree, disagree, strongly disagree

Are you aware of the criteria for the diagnosis of PCOS? Yes/No

Reflecting on your symptoms and signs of PCOS, how many years earlier do you think you could have been diagnosed with PCOS? (answer in numbers

Irregular periods

My clinicians are confident in managing the menstruation-related issue in women PCOS- strongly agree, agree, neither agree or disagree, disagree, strongly disagree

I am aware of the options available for management of menstruation -related issue in women with PCOS - strongly agree, agree, neither agree or disagree, disagree, strongly disagree

Skin

My clinicians are confident in managing the skin-related issue in women PCOS- strongly agree, agree, neither agree or disagree, disagree, strongly disagree

I am aware of the options available for management of skin-related issue in women with PCOS - strongly agree, agree, neither agree or disagree, disagree, strongly disagree

Weight

My clinicians are confident in managing the weight-related issue in women PCOS

- strongly agree, agree, neither agree or disagree, disagree, strongly disagree

I am aware of the options available for management of weight-related issue in women with PCOS - strongly agree, agree, neither agree or disagree, disagree, strongly disagree

Fertility

My clinicians are confident in managing the fertility-related issue in women PCOS- strongly agree, agree, neither agree or disagree, disagree, strongly disagree

I am aware of the options available for management of fertility-related issue in women with PCOS - strongly agree, agree, neither agree or disagree, disagree, strongly disagree

**Supplementary 6B.** Example of Post-SIMBA-PPI survey for patients.

**Post-SIMBA-PCOS survey (Patients)**

Acceptance and session feedback

I think SIMBA-PCOS will benefit other women with PCOS understand more about this condition - strongly agree, agree, neither agree or disagree, disagree, strongly disagree

I think SIMBA-PCOS helped bridge the gap of expectations between clinicians and women with PCOS - strongly agree, agree, neither agree or disagree, disagree, strongly disagree

I think SIMBA-PCOS will improve the overall experience in the management of women with PCOS

- strongly agree, agree, neither agree or disagree, disagree, strongly disagree

Please provide us feedback to help us improve for upcoming sessions (free-text)

Diagnosis

I am satisfied with my diagnosis experience of PCOS- strongly agree, agree, neither agree or disagree, disagree, strongly disagree

My clinician(s) provided myself with adequate information on PCOS at diagnosis- strongly agree, agree, neither agree or disagree, disagree, strongly disagree

Are you aware of the criteria for the diagnosis of PCOS? Yes/No

Reflecting on your symptoms and signs of PCOS, how many years earlier do you think you could have been diagnosed with PCOS? (answer in numbers

Fertility

My clinicians are confident in managing the fertility-related issue in women PCOS- strongly agree, agree, neither agree or disagree, disagree, strongly disagree

I am aware of the options available for management of fertility-related issue in women with PCOS - strongly agree, agree, neither agree or disagree, disagree, strongly disagree

Weight

My clinicians are confident in managing the weight-related issue in women PCOS

- strongly agree, agree, neither agree or disagree, disagree, strongly disagree

I am aware of the options available for management of weight-related issue in women with PCOS - strongly agree, agree, neither agree or disagree, disagree, strongly disagree

Skin

My clinicians are confident in managing the skin-related issue in women PCOS- strongly agree, agree, neither agree or disagree, disagree, strongly disagree

I am aware of the options available for management of skin-related issue in women with PCOS - strongly agree, agree, neither agree or disagree, disagree, strongly disagree

Irregular periods

My clinicians are confident in managing the menstruation-related issue in women PCOS- strongly agree, agree, neither agree or disagree, disagree, strongly disagree

I am aware of the options available for management of menstruation -related issue in women with PCOS - strongly agree, agree, neither agree or disagree, disagree, strongly disagree

**Supplementary 6C.** Example of Pre-SIMBA-PPI survey for HCPs.

**Pre-SIMBA-PCOS survey (HCPs)**

I consent voluntarily to be a participant in this study and understand that I can
refuse to answer questions and I can withdraw from the study at any time,
without having to give a reason, up until the online survey is submitted; and that

after the survey is submitted it will not be possible to withdraw (as it will not be

possible to link you personally with the information submitted online).

(Yes/No)

Diagnosis

I think most women with PCOS are satisfied with their diagnosis experience of PCOS- strongly agree, agree, neither agree or disagree, disagree, strongly disagree

I always provide my patient with adequate information on PCOS at diagnosis- strongly agree, agree, neither agree or disagree, disagree, strongly disagree

I think women with PCOS could be diagnosed earlier with more awareness to this condition - strongly agree, agree, neither agree or disagree, disagree, strongly disagree

Please comment on what you think are the barriers for delayed diagnoses: (free text)

Fertility

I am confident in managing the fertility-related issue in women PCOS- strongly agree, agree, neither agree or disagree, disagree, strongly disagree

Weight

I am confident in managing the weight-related issue in women PCOS

- strongly agree, agree, neither agree or disagree, disagree, strongly disagree

Skin

I am confident in managing the skin-related issue in women PCOS- strongly agree, agree, neither agree or disagree, disagree, strongly disagree

Irregular periods

I am confident in managing the menstruation-related issue in women PCOS- strongly agree, agree, neither agree or disagree, disagree, strongly disagree

**Supplementary 6D.** Example of Post-SIMBA-PPI survey for HCPs.

**Post-SIMBA-PCOS survey (HCPs)**

Acceptance and session feedback

I think SIMBA-PCOS will benefit other women with PCOS understand more about this condition - strongly agree, agree, neither agree or disagree, disagree, strongly disagree

I think SIMBA-PCOS-helped bridge the gap of expectations between clinicians and women with PCOS- strongly agree, agree, neither agree or disagree, disagree, strongly disagree

I think SIMBA-PCOS will improve the overall experience in the management of women with PCOS

- strongly agree, agree, neither agree or disagree, disagree, strongly disagree

Please provide us feedback to help us improve for upcoming sessions (free-text)

Diagnosis

I think most women with PCOS are satisfied with their diagnosis experience of PCOS- strongly agree, agree, neither agree or disagree, disagree, strongly disagree

I always provide my patient with adequate information on PCOS at diagnosis- strongly agree, agree, neither agree or disagree, disagree, strongly disagree

I think women with PCOS could be diagnosed earlier with more awareness to this condition - strongly agree, agree, neither agree or disagree, disagree, strongly disagree

Fertility

I am confident in managing the fertility-related issue in women PCOS- strongly agree, agree, neither agree or disagree, disagree, strongly disagree

Weight

I am confident in managing the weight-related issue in women PCOS

- strongly agree, agree, neither agree or disagree, disagree, strongly disagree

Skin

I am confident in managing the skin-related issue in women PCOS- strongly agree, agree, neither agree or disagree, disagree, strongly disagree

Irregular periods

I am confident in managing the menstruation-related issue in women PCOS- strongly agree, agree, neither agree or disagree, disagree, strongly disagree

**Supplementary 7.** Demographics of HCP attending SIMBA-PCOS and SIMBA-Adrenal.

|  | SIMBA-PCOS: Frequency, n/N | SIMBA-Adrenal: Frequency, n/N |
| --- | --- | --- |
| Country of Origin |  |  |
| UK | 13/25 | 10/23 |
| Non-UK | 12/25 | 13/23 |
| Training Level |  |  |
| Specialist | 2/25 | 10/23 |
| Specialist in training | 15/25 | 15/23 |
| Non-specialist | 6/25 | 1/23 |
| Other | 2/25 | 1/23 |

**Supplementary 8.** Demographics of people with PCOS attending SIMBA-PCOS.

|  | **Frequency, n/N** |
| --- | --- |
| Country of origin |  |
| UK | 9/15 |
| Non-UK | 6/15 |
| Ethnicity |  |
| White | 9/15 |
| Asian | 5/15 |
| Black | 1/15 |
| Age of diagnosis (years) |  |
| 0-10 | 2/15 |
| 11-20 | 8/15 |
| 21-30 | 5/15 |
